# Supplementary material for: Traditional Human Immunodeficiency Virus treatment and family and social influence as barriers to accessing HIV care services in Belu, Indonesia
Source: PLoS One. 2022 Jul 25;17(7):e0264462. doi: 10.1371/journal.pone.0264462 (PMC9312407; doi:10.1371/journal.pone.0264462)
Supplement: Attachment — Submitted filename: Response to Reviewers.docx [file pone.0264462.s002.docx]

**Response to Reviewers**

Dear Editor,

Thank you for considering our paper for publication in **PLOS ONE**. Herewith we submit the revised version of our paper entitled: **“Traditional Human Immunodeficiency Virus treatment and family and social influence as barriers to accessing HIV care services in Belu, Indonesia”**. We have improved the manuscript and addressed each of the comments from the reviewer as reported in the ‘a point-by-point response’ below.

On behalf of all authors,

Sincerely,

Nelsensius Klau Fauk

**Journal Requirements:**

When submitting your revision, we need you to address these additional requirements.

1. Please ensure that your manuscript meets PLOS ONE's style requirements, including those for file naming. The PLOS ONE style templates can be found at

<https://journals.plos.org/plosone/s/file?id=wjVg/PLOSOne_formatting_sample_main_body.pdf>  and <https://journals.plos.org/plosone/s/file?id=ba62/PLOSOne_formatting_sample_title_authors_affiliations.pdf>

***Response***:

- Yes, the guidelines have been followed.

2. Please include a complete copy of PLOS’ questionnaire on inclusivity in global research in your revised manuscript. Our policy for research in this area aims to improve transparency in the reporting of research performed outside of researchers’ own country or community. The policy applies to researchers who have travelled to a different country to conduct research, research with Indigenous populations or their lands, and research on cultural artefacts. The questionnaire can also be requested at the journal’s discretion for any other submissions, even if these conditions are not met.  Please find more information on the policy and a link to download a blank copy of the questionnaire here: https://journals.plos.org/plosone/s/best-practices-in-research-reporting. Please upload a completed version of your questionnaire as Supporting Information when you resubmit your manuscript.

***Response***:

- PLOS’ questionnaire on inclusivity in global research is attached.
- We would like to explain that the corresponding author (NKF) is from Belu district, Indonesia, where study was conducted. He was the field researcher who conducted the interviews with all the participants as reported in pages 6-8.

3. In your Data Availability statement, you have not specified where the minimal data set underlying the results described in your manuscript can be found. PLOS defines a study's minimal data set as the underlying data used to reach the conclusions drawn in the manuscript and any additional data required to replicate the reported study findings in their entirety. All PLOS journals require that the minimal data set be made fully available. For more information about our data policy, please see http://journals.plos.org/plosone/s/data-availability.

Upon re-submitting your revised manuscript, please upload your study’s minimal underlying data set as either Supporting Information files or to a stable, public repository and include the relevant URLs, DOIs, or accession numbers within your revised cover letter. For a list of acceptable repositories, please see http://journals.plos.org/plosone/s/data-availability#loc-recommended-repositories. Any potentially identifying patient information must be fully anonymized.

Important: If there are ethical or legal restrictions to sharing your data publicly, please explain these restrictions in detail. Please see our guidelines for more information on what we consider unacceptable restrictions to publicly sharing data: http://journals.plos.org/plosone/s/data-availability#loc-unacceptable-data-access-restrictions. Note that it is not acceptable for the authors to be the sole named individuals responsible for ensuring data access.

We will update your Data Availability statement to reflect the information you provide in your cover letter.

***Response***:

- Data availability statement:

We would like to explain that the data cannot be shared publicly because of restriction set by the human research ethics committees. Data presented in this study are available on request from the ethics committee imposing the restriction upon the data or from the corresponding author for researchers who meet the criteria for access to confidential data.

Our data in this study comprise transcriptions of digital audio recordings of each individual interview with women and men living with HIV and healthcare professionals in Belu district, Indonesia. The interviews were focused on exploring their views and experiences about HIV-risk factors, their lived experiences of HIV impacts and barriers to access to HIV care services.

The nature of the restrictions upon the data is ethical due to the reason that participants (women and men living with HIV) involved in this study are considered by the ethics committee as highly vulnerable groups and data obtained from them may contain sensitive information.

The full name of the ethics committee imposing the restrictions upon the data is Social and Behavioural Research Ethics Committee, Flinders University, South Australia, Australia. For more information regarding ethical approval of the project please contact the Executive Officer on +618 8201-3116 or [human.researchethics@flinders.edu.au](mailto:human.researchethics@flinders.edu.au)

**Comments to the Author**

**Reviewer** #1: Traditional treatment of HIV and the role of family members as barriers to access to HIV care service or antiretroviral therapy among people living with HIV

Use of abbreviations like HIV in the title is not recommended and the title is not well articulated.
- Possibly “Traditional treatment of Human Immunodeficiency Virus as barriers to access HIV care and treatment in Belu, Indonesia”.
- Still can well articulate the title…

***Response***:

- Title has been revised:

Traditional Human Immunodeficiency Virus treatment and family and social influence as barriers to accessing HIV care services in Belu, Indonesia

**General**: The paper raised an important issue that is the use of traditional treatments in Belu is a barrier to HIV care and ART. The role players for decision-makers for use of traditional medicine (TM) were family members and the culture. The impact of this practice is to the extent that the patients loss their life. However, this important finding is not well written and elaborated to show how much the use of traditional medicine in these specific areas is affecting the life of HIV patients. The paper is poorly written, I recommend this better be rewritten clearly organizing the drivers for the use TM and its impacts on the life of patients. In writing the manuscript please avoid unnecessary long statements (some of which seems desire to extend the statement – better use short sentences)
- It would have been better if the impact on traditional medicine use on access to biomedical HIV care & treatment was assessed.
- Lacks the list of abbreviations/acronyms
- Good to have operational definitions for some terminologies. (like TM, traditional healers … in your context)

***Response***:

- We have elaborated the impact of the practice of traditional medicine use for HIV treatments on health seeking behaviours of PLHIV, their access to HIV care and treatment services, their health condition and economic condition (see Results Section).
- The manuscript has been edited and revised. All unnecessary long and ambiguous sentences have been improved and shortened.
- List of abbreviations/acronyms have been provided underneath the Conclusions Section

AIDS	: Acquired immune deficiency syndrome

ART	: Antiretroviral therapy

HCPs	: Healthcare professionals

HIV	: Human Immunodeficiency Virus

PLHIV	: People living with HIV

THs	: Traditional healers

TMs	: Traditional medicines

- Operational definitions for terminologies such as traditional medicine, traditional healer have been provided at the first time each of them was used.

Despite a range of barriers as reported in the aforementioned studies, the literature suggests that evidence on the influence of cultural practices (e.g., the use of traditional medicines (TMs)), family and social factors on access to HIV care services among PLHIV is still limited. TMs in the context of Belu refer to the roots, leaves and barks of plants that are prepared and mixed by traditional healers (THs) or family members and used for the treatment of HIV in PLHIV. THs in this context refer to people who do not attend any formal medical training but are considered by family or community members as being competent to provide HIV care using roots, leaves and barks of plants based on traditional practices.

**Abstract**:
- “As a part of a qualitative study in Belu, this paper describes the use of traditional treatment and the role of families in determining traditional treatment for their HIV-positive family member as barriers to access to HIV care service or ART among PLHIV.” This statement not clear better to rewrite in more clear way.

***Response***:

- As a part of a qualitative study in Belu, this paper describes the use of traditional medicines for HIV treatment and family and social influence as barriers to access to HIV care services among PLHIV.

- Snow ball technique you used is not clear – what is the information pack?

***Response***:

- One-on-one in-depth interviews were employed to collect data from 46 PLHIV (26 women and 20 men) and 10 healthcare professionals. They were recruited using the snowball sampling technique. The study information sheets were initially posted on information boards in healthcare facilities. Potential participants who contacted to confirm their participation were recruited for an interview and then asked for help to distribute the information sheets to their eligible colleagues who might be willing to participate.

**Background**
- Good coverage, however most sentences are too long that need to be rephrased.
***Response***:

- All long sentences throughout the manuscript have been revised.

Last paragraph: In this aim statement and throughout the manuscript you have used terms in different words like “HIV care services or ART”, “traditional treatment or medicines” which you could use either of them or better expressive phrase.

***Response***:

- Revisions to the use of these terms have been made throughout the manuscript. Mostly term HIV care services and traditional medicines are now used throughout the manuscript.

- Why you did not discuss on the barriers to access ART, rather than having too narrow scope that focus only on family impact.

***Response***:

- This paper aimed to fill this gap in knowledge by exploring cultural practice of the use of TMs for HIV treatments in Belu, and family and social influence towards HIV treatment of PLHIV. It also explored how these barriers to HIV care access had impacted health outcomes and life of PLHIV.

**Methods**:
Study Setting:
Recruitment and Data Collection:
P1. “As part of a larger qualitative study to understand HIV risk factors, impacts and determinants of access to HIV care services among women and men living with HIV in Belu, this paper describes the use of traditional treatment for HIV and the role of family members, friends and neighbours in supporting the use of traditional treatment as barriers to the access of PLHIV to HIV care service or ART”.
- The aim of this study was already described in the background section; not necessary to repeat it here (remove this statement).

***Response***:

- The statement has been removed.

The snowball technique used is not clear. I do not think this is a snowball technique? What was the study information pack given to the patients and HCPs? “HIV clinic receptionist and healthcare facilities” not clear.

***Response***:

- It is the snowball sampling technique. Revision has been made to clarify the above comments:

The participants were PLHIV and healthcare professionals (HCPs) recruited using the snowball sampling technique. PLHIV and HCPs were respectively recruited from an HIV clinic and public health centres (Puskesmas) providing HIV care services in Belu. Following the initial talk and agreement with the heads of these healthcare facilities, the study information sheets for PLHIV and HCPs were initially distributed through the receptionists in these healthcare facilities. The information sheets which contained a brief explanation about the study and contact details of the field researcher (NKF) were posted by the receptionists on their information boards. Participants who called to confirm their participation were recruited and asked to suggest a preferred time and place for an interview. The initially interviewed participants were also asked for help to distribute the study information sheets to their eligible friends and colleagues who might be willing to participate. This process was iterative, leading to a total of 46 PLHIV (26 women and 20 men) and 10 HCPs participating in this study.

P1L11: “The initially interviewed participants were also asked to distribute the study information packs to their friends and colleagues who might be willing to participate.”
- I think this information sharing between participants makes the second group of participants affected (influenced) by others resulting in biased information.

***Response***:

- This has been revised as following:

The initially interviewed participants were also asked for help to distribute the study information sheets to their eligible friends and colleagues who might be willing to participate. This process was iterative, leading to a total of 46 PLHIV (26 women and 20 men) and 10 HCPs participating in this study.

The method section should have clearly shown the positionality of the researcher (criticality), validity… quality measure of a qualitative study.

Response:

- The method section has been revised.

**Result**:
Generally good. Rephrase the statements to sound scientific.
• Table 1: The occupation for HCPs and PLHIV need to be separate. Also, the list of occupation needs to be re-categorized.
***Response***:

- Occupation for HCPs and PLHIV has been separated and the list of occupation has been re-categorised.

**Discussion**:
Almost the result is rewritten. It is better to make well-referenced and explained comparing with national or international policies and research findings.

***Response***:

- The entire Discussion Section has been revised and well-referenced. Comparisons of our findings with previous findings globally have also been provided.

P1L1: different form referencing (UNAIDS 2020). Make uniform.

***Response***:

- This referencing style has been corrected.

**Reviewer** #2: This was an interesting article. There are some aspects that require revision. First, the entire paper needs to be professionally edited for English language. Many of the sentences are long and the english is hard to follow.

***Response***:

- The entire manuscript has been edited and revised.

**Introduction**: I recommend describing barriers to use of ART rather than barriers to accessing HIV care services or use of ART- this is just too wordy and hard to follow.

***Response***:

- This has been addressed throughout the manuscript and the term ‘barriers to accessing HIV care services’ is now used as it is the focus of this study.

Please describe the role and use of traditional medicines in Indonesia- more information is needed on the context of these medicines and how traditional and western systems of care for HIV interact if at all.

***Response***:

- The use of TMs for treatment of any health issues, including HIV, is also a common practice within families and communities in this area, which might have been a barrier to the access of PLHIV to HIV care services. To the best of our knowledge, there has not been any study exploring the influence of TMs and the social influence of families and friends on access to HIV care services among PLHIV in the context of Belu and Indonesia. Belu was selected as the study setting due to feasibility, familiarity and the potential of undertaking the current study successfully.

**Methods**: The sampling approach you mention here is not snowball sampling- rather convenience sampling

***Response***:

- It is the snowball sampling technique. Revision has been made to make it clear:

The participants were PLHIV and healthcare professionals (HCPs) recruited using the snowball sampling technique. PLHIV and HCPs were respectively recruited from an HIV clinic and public health centres (Puskesmas) providing HIV care services in Belu. Following the initial talk and agreement with the heads of these healthcare facilities, the study information sheets for PLHIV and HCPs were initially distributed through the receptionists in these healthcare facilities. The information sheets which contained a brief explanation about the study and contact details of the field researcher (NKF) were posted by the receptionists on their information boards. Participants who called to confirm their participation were recruited and asked to suggest a preferred time and place for an interview. The initially interviewed participants were also asked for help to distribute the study information sheets to their eligible friends and colleagues who might be willing to participate. This process was iterative, leading to a total of 46 PLHIV (26 women and 20 men) and 10 HCPs participating in this study.

Data analysis section- it very long and can be abbreviated

***Response***:

- Data analysis section has been shortened:

The audio recordings were transcribed verbatim and manually using a laptop by the first author (NKF). Data analysis was performed using NVivo 12 software and guided by the five steps introduced in qualitative data analysis framework by Ritchie and Spencer. These steps are (i) familiarisation with the data or transcripts through reading the transcripts line by line and repeatedly, providing comments and labels, and highlighting ideas related to barriers to access to HIV care services; (ii) identification of a thematic framework by writing down key issues and concepts, (iii) indexing the data by creating open coding to each individual transcript, where data extracts of each transcript were given a code or node. This was followed by close coding to identify similar or redundant codes and group them together to reduce the length of the coding list. Codes that formed the same themes or sub-themes were grouped together; (iv) charting data by arranging a thematic framework in a summary of chart; and (v) mapping and interpretation of the data. Data analysis was primarily performed by the field researcher, although team-based analysis was carried out at regular supervision meetings whereby comments and suggestions were provided and discussed and then team decisions were made about the validity of the final themes and interpretation.

All the COREQ guidelines have not been followed- please check and report back for example on member checking

***Response***:

- The method section has been revised to make sure that all the items in the COREQ checklist are reported.

For example:

Data analysis was primarily performed by the field researcher, although team-based analysis was carried out at regular supervision meetings whereby comments and suggestions were provided and discussed and then team decisions were made about the validity of the final themes and interpretation.

**Results**: You only need 1-2 illustrative quotes per section. Three is too many. There seems to be some overlap between the themes - eg family influence permeates across several themes

***Response***:

- The result section has been improved.
- Only 1-2 quotes are used per section.

**Discussion**. This needs the most work. You report the findings but do not discuss the implications for policy and practice. If family is so influential what can be done to improve HIV health literacy, overcome medication myths etc, can traditional healers be engaged in the HIV care system to advocate for use of ART? Needs to move beyond mere reporting of the results.

***Response***:

- The entire discussion section has been revised and improved. The implications of the findings for HIV-related policies and practice to address the needs of PLHIV and their families, and community members have been provided. (See Discussion Section).
